# Neuronal Representation of Locomotion During Motivated Behavior in the Mouse Anterior Cingulate Cortex

**DOI:** 10.3389/fnsys.2021.655110

**Published:** 2021-04-29

**Authors:** Sachuriga, Hiroshi Nishimaru, Yusaku Takamura, Jumpei Matsumoto, Mariana Ferreira Pereira de Araújo, Taketoshi Ono, Hisao Nishijo

**Affiliations:** ^1^System Emotional Science, Faculty of Medicine, University of Toyama, Toyama, Japan; ^2^Graduate School of Innovative Life Science, University of Toyama, Toyama, Japan; ^3^Research Center for Idling Brain Science, University of Toyama, Toyama, Japan; ^4^Department of Physiological Sciences, Health Sciences Center, Federal University of Espirito Santo, Vitoria, Brazil

**Keywords:** prefrontal cortex, motor control, goal-directed behavior, reward, locomotion, mouse model

## Abstract

The anterior cingulate cortex (ACC) is located within the dorsomedial prefrontal cortex (PFC), and processes and facilitates goal-directed behaviors relating to emotion, reward, and motor control. However, it is unclear how ACC neurons dynamically encode motivated behavior during locomotion. In this study, we examined how information for locomotion and behavioral outcomes is temporally represented by individual and ensembles of ACC neurons in mice during a self-paced locomotor reward-based task. By recording and analyzing the activity of ACC neurons with a microdrive tetrode array while the mouse performed the locomotor task, we found that more than two-fifths of the neurons showed phasic activity relating to locomotion or the reward behavior. Some of these neurons showed significant differences in their firing rate depending on the behavioral outcome. Furthermore, by applying a demixed principal component analysis, the ACC population activity was decomposed into components representing locomotion and the previous/future outcome. These results indicated that ACC neurons dynamically integrate motor and behavioral inputs during goal-directed behaviors.

## Introduction

It is widely accepted that emotion affects movement. When we engage in a continuous and/or repetitive reward-seeking activity that involves movement, we make unconscious decisions based on our past experiences and potential outcomes to value the effort. In many mammals, including rodents, such an activity involves overground locomotion (e.g., walking and running), which allows the animal to move from one location to another. The locomotor centers are widely distributed throughout the central nervous system from the forebrain to the spinal cord. To date, many of the supraspinal structures, such as the brainstem, hypothalamus, and basal ganglia, have been extensively studied (for recent reviews, see Ferreira-Pinto et al., 2018; Grillner and El Manira, 2020). However, aside from the sensorimotor cortex and the posterior parietal cortex, the role of many cortical areas controlling and modulating locomotion remains unraveled (Drew and Marigold, 2015).

The prefrontal cortex (PFC) is implicated in the decision-making process, value coding, and controlling movement during goal-directed behaviors (Sul et al., 2010; Fuster, 2015). The anterior cingulate cortex (ACC) lies within the dorsomedial PFC and is involved in motor control, sensory processing, emotion, motivation, and evaluation of outcome value (for reviews see Bush et al., 2000; Allman et al., 2006; Rushworth and Behrens, 2008; Euston and McNaughton, 2006). The ACC has outputs to the motor cortex and spinal cord (Gabbott et al., 2005; Hoover and Vertes, 2007), suggesting a role in motor planning and execution (Dum and Strick, 1991; Devinsky et al., 1995). In humans, it has been reported that damage to the ACC causes motor deficits such as akinetic mutism or impairments in movement initiation (Mesulam, 1981; Devinsky et al., 1995). Moreover, monkeys with ACC lesions or chemical inactivation of the ACC show impaired performance for self-paced motor tasks (Thaler et al., 1995; Shima and Tanji, 1998). Despite this evidence, the role of the ACC in controlling locomotion is still unclear.

The ACC is also implicated in behavioral monitoring. It has been shown in monkeys that ACC neurons encode the behavioral outcome while the animal is engaged in a task to obtain rewards (Ito et al., 2003; Matsumoto et al., 2003; Kennerley et al., 2009; Hayden and Platt, 2010). The ACC is strongly connected with dopaminergic centers, namely the ventral tegmental area (VTA) (Williams and Goldman-Rakic, 1993; Carr and Sesack, 2000); thus it is assumed to integrate emotion and motion (for a recent review, see Rolls, 2019). In rodents, ACC activity is modulated by emotional and motivational inputs during goal-directed behavior, such as reward value during navigation (Euston and McNaughton, 2006; Cowen et al., 2012). However, it is unknown how the neurons dynamically encode locomotor information while integrating such inputs. In this study, we recorded and analyzed the activity of ACC neurons in mice during a self-paced locomotor reward task. We show that a significant number of neurons encoded the initiation and termination of locomotion as well as the reward-related information during the task. Moreover, by examining the ensemble activity of these neurons, we show that ACC activity primarily represents the time course of locomotion together with the past and present behavioral outcome.

## Materials and Methods

### Subjects

Seven male C57BL/6J mice (25–30 g; Japan SLC, Inc., Hamamatsu, Japan) were used in this study. We used male mice for this study to rule out the possible effect of estrous cycle-dependent modulation of PFC activity in female mice (Galvin and Ninan, 2014). All mice were treated in compliance with the guidelines for the Care and Use of Laboratory Animals at the University of Toyama. The procedures in this study were approved by the Ethical Committee of Animal Experiments at the University of Toyama (Permit No. A2017MED-18, A2020MED-9).

### Surgical Procedures

Mice were deeply anesthetized with a combination of 0.3 mg/kg medetomidine, 4.0 mg/kg midazolam, and 5.0 mg/kg butorphanol by intraperitoneal injection. All surgical procedures were performed under stereotaxic control, thermoregulation, and deep anesthesia. Briefly, the mouse head was fixed in a stereotaxic apparatus (Narishige, Tokyo, Japan). Body temperature was maintained with a heating pad placed under the animal. To record neural signals, a single array with four tetrodes (groups of four twisted 13 μm tungsten wires, 280 μm apart, 200–500 kΩ, California Fine Wire, Grover Beach, CA) was implanted in the left PFC (according to the Franklin and Paxinos (2008) coordinates: X +0.5 mm, Y 0.0 mm to +1.0 mm, Z −1.2 mm) using a custom microdrive allowing for the adjustment of individual tetrodes. The microdrive was implanted into the left PFC due to spatial restriction of the head fixing apparatus. A stainless steel screw was implanted above the right cerebellum to serve as a ground. The microdrive was fixed to the skull with dental cement (Super-Bond C&B, Sun Medical, Shiga, Japan). An aluminum frame (CF-10, Narishige) was cemented on the exposed skull for the animal to be attached to the behavioral apparatus. After the surgery, a non-steroidal anti-inflammatory drug (2 mg/kg meloxicam) was administered by subcutaneous injection, and antibiotic (gentamicin sulfate) was administered topically. Following recovery from anesthesia, mice were returned to their cage and housed individually. They were given at least 1 week of recovery with careful monitoring of body weight before the behavioral training started.

### Behavioral Apparatus and Task

Mice were trained to perform a self-paced locomotor task consisting of repeated runs on a spherical treadmill to obtain a reward. After recovery from the surgery, mice were first familiarized with the behavioral apparatus for 3–4 days prior to training. The mice were water-deprived for 12 h in their cages before the training and recording sessions.

**FIGURE 1:**
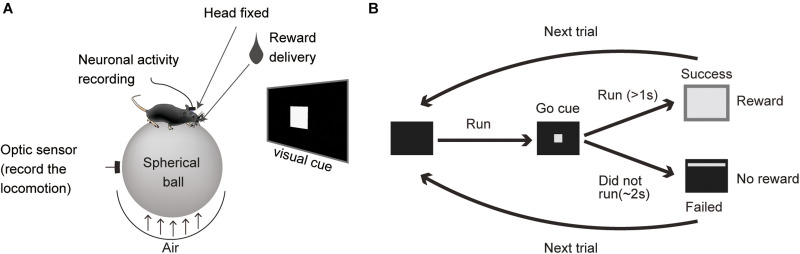
Locomotor task. **(A)** A schematic for the task apparatus. **(B)** Diagram of the task sequence.

The mice were trained to run on a spherical Styrofoam ball with their head fixed to a custom-made column (Figure 1A). A 19 inch LCD monitor was placed in front of the mouse during training and recording sessions. It was placed 30 cm away at eye-level for the secured mouse. The locomotor task sequence was programmed and run using E-prime software (Psychology Software Tools, Inc., Sharpsburg. PA). A white square (5 × 5 cm) was presented as a visual cue. The reward (4 μl drop of sucrose water) was dispensed from the tip of a spout that was placed in front of their mouth by a micro-pump (water supply unit, O’Hara, Tokyo, Japan). During the task, the mouse had to initiate a run and then stop (preparatory run). In every trial, when the mouse stopped, the visual cue was presented for 2 s, a time window in which the animal had to restart running (reward run). After running for more than 1 s, sucrose water was dispensed as a reward (Figures 1A,B) and a white rectangle (35 × 30 cm) was presented for 4 s as visual feedback for rewarded trials. If the mouse did not restart or complete the 1 s reward run, the visual cue was turned off, they were not rewarded (non-reward trial) and had a 3 s time out (the cue cannot be triggered during this period).

The speed of the ball movement was measured with an optical sensor positioned in front of it. Before the first session, the position of the ball was finely tuned for each mouse to align with the direction of the locomotion, which remained unchanged until the final session of the day. The X–Y coordinate data from the optical mouse were fed into an Arduino micro-controller that computed the velocity of the ball.

After the mice learned to run on the treadmill, they were trained to perform 105–120 trials divided into seven or eight sessions (15 trials per session) in 1 day. After 2–3 weeks of training, the mice successfully received a reward in more than 60% of the trials in at least two out of seven sessions. Over the next 3–4 weeks, the locomotor activity, behavioral outcomes (rate of the reward), and neuronal ACC activity were recorded.

### Neuronal and Behavioral Recording

ACC electrophysiological activity was recorded by the tetrode array while the mice performed the self-paced locomotor task described above. The positions of the tetrodes were lowered at >100 μm intervals after each recording day to obtain maximal unit detection in the ACC and sample from a wide-range of depths.

The amplified neuronal signals were digitized at a 40 kHz sampling rate, and 0.8 ms waveforms that crossed an experimenter-defined threshold were stored with a Multichannel Acquisition Processor system (MAP: Plexon Inc., Dallas, TX) on a computer disk for offline spike analysis. The locomotor velocity, timing of the cue signals, reward run initiation, and reward delivery were digitized at a 40 kHz sampling rate and stored on a computer disk.

The behavior of the mice on the ball was captured at 30 frames/s by a charged-coupled device (CCD) camera. Data were stored on a hard disk using the CinePlex program (Plexon Inc.), which synchronized the video images with the neuronal data. The video was captured obliquely from the side of the apparatus.

### Histology

**FIGURE 2:**
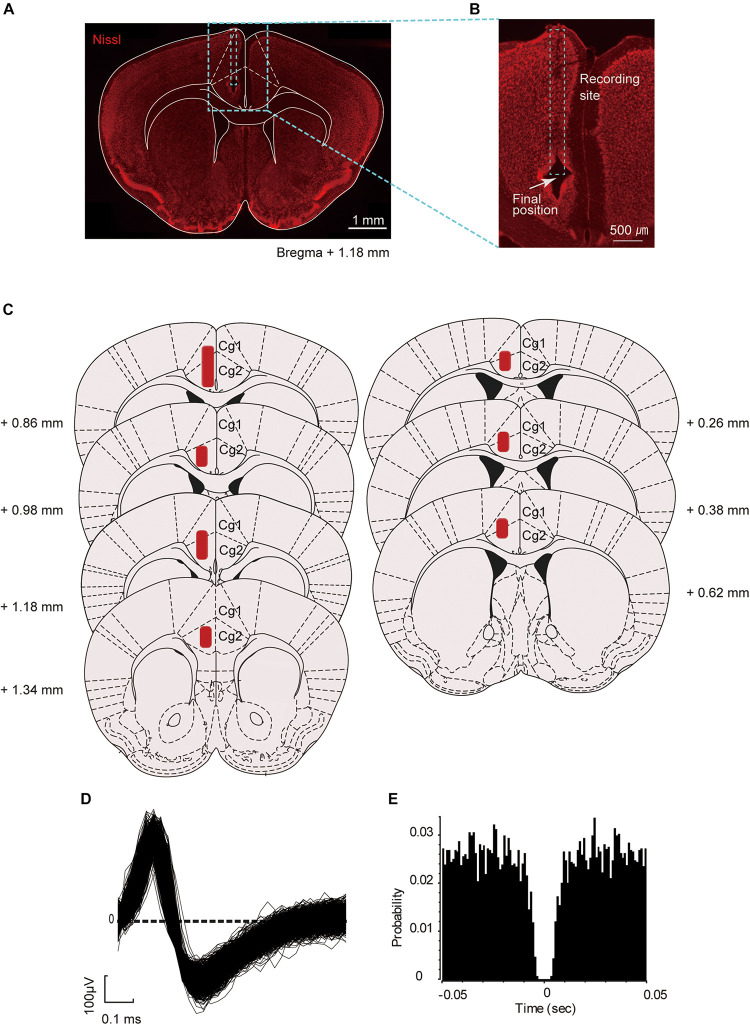
Location of the recording sites and unit waveform. **(A)** Representative coronal section of the prefrontal cortex (PFC) of the mouse brain (Nissl stained) targeted for electrophysiological recordings. **(B)** Position of the electrolytic lesion after the last recording. **(C)** Schematic coronal sections summarizing the positions of the tetrodes in the PFC (from seven mice). The area in red indicates the putative recording site. The positions of the tetrode were projected onto plates from Franklin and Paxinos (2008) with permission. **(D)** An example of superimposed spike waveforms of an ACC neuron. **(E)** Autocorrelogram of the neuron shown in **(D)**, with the bin of 1 ms. Ordinates indicate probability, where bin counts were divided by the number of spikes in the spike train.

After the final electrophysiology experiment, the mice were deeply anesthetized, and tetrode positions were marked by electrolytic lesioning of brain tissue (30 μA negative current for 7 s through each electrode). The mice were perfused transcardially with 0.9% saline followed by 4% paraformaldehyde (PFA). Brains were extracted, fixed in PFA for 24 h, and sectioned (coronal, 50 μm thickness) on a microtome. The sections were stained with fluorescent Nissl (NeuroTrace, Thermo Fisher Scientific, Waltham, MA) and DAPI (Vector Laboratories, Burlingame, CA), mounted using Vectashield (Vector Laboratories), and then visualized with a fluorescent microscope (Figures 2A,B). To confirm the final position of the tetrodes, the identified recording sites were re-plotted according to the corresponding sections of the mouse brain atlas reported by Franklin and Paxinos (2008; Figure 2C).

### Single Unit Isolation and Classification

The digitized neuronal activity was isolated into single units by waveform components in a similar way to that described in our previous study (Matsumoto et al., 2012). Briefly, spike sorting was performed with the Offline Sorter program (Plexon Inc.) by manually clustering single units of the four electrodes and plotting the waveform data as three-dimensional projections. For each isolated cluster, an interspike interval histogram was constructed, and only units with an absolute refractory period of at least 2 ms were included in the data analysis (Figures 2D,E). Superimposed waveforms of the isolated units were drawn to check their consistency throughout the seven recording sessions. Only units that were stably recorded throughout the seven sessions were included for analysis. Furthermore, those that did not fire within −2.0 s to +2.0 s around the key task events were excluded from the analysis. The key task events were categorized as start of the preparatory run (start), visual-cue presentation (cue), and reward delivery (reward).

The isolated units were classified into wide-spike (WS) putative pyramidal neurons and narrow-spike (NS) putative interneurons based on the distribution of (1) the peak-to-valley ratio (the ratio between the amplitude of the initial peak and the following trough) and (2) the half-valley width of each spike waveform. Each unit was classified based on the distribution calculated by fitting a Gaussian mixture model (GMM) (Stark et al., 2013; Kim et al., 2016) using the *fitgmdist* function in MATLAB.

### Neural Correlates to the Locomotor Task

All data analysis was performed using neuroexplorer (Plexon Inc.) and MATLAB (Mathworks). To identify which ACC neurons were active during the locomotor task, the data were plotted in a peri-event histogram (PETH) and smoothed with a Gaussian filter (σ = 100 ms). Phasic activity change during the peri-events was analyzed with a repeated one-way ANOVA and *post hoc* Bonferroni test (*p* < 0.05 for statistical significance). The neurons that significantly increased their firing rate around the reward event were defined as Type 1 neurons. Neurons that showed significantly higher and lower spike rate in the post-start (+1.5 s from the start) compared with the pre-start (−1.5 s from the start) period were defined as Type 2 neurons and Type 3 neurons, respectively.

The *Z*-scored firing rate was calculated from the instantaneous firing rate for each unit, which was produced by convolving a Gaussian kernel with σ = 50 ms resampled at 100 Hz for each single trial. Pearson’s correlations covariance (r) between the firing rate of individual units and running velocity were calculated −1.5 s prior to the start to the cue presentation, in time bins of 1 s. Units with Pearson’s *r*-value > 0.3 or < −0.3 and *p* < 0.05 were considered significantly correlated with the running velocity.

The differences in neural response between different behavioral outcomes were analyzed by comparing PETHs in rewarded and unrewarded trials around the key events using the permutation test proposed in Fujisawa et al., 2008, implemented in FMAToolbox^1^ for MATLAB. Briefly, for the statistical test, first the difference *D_0_(t)* between the PETHs were computed as:

*D_0_ (t) = f_R_(t) − f_U_(t)*

where, *f_R_(t)* and *f_U_(t)* represent smoothed PETH (bin size = 250 ms) with a Gaussian filter (*σ* = 50 ms). Secondly, a difference was similarly computed with a shuffled data in which the labels (rewarded/unrewarded) of trials were randomly permutated. This process was repeated *M* times (*M* = 5000) to obtain the differences of resampled data, *D_1_(t),…, D_M_(t)*. Then, for a given time *t*, *p*-value, *p(t)* was computed:

*p(t) = min{1, 2p^+^(t), 2p^–^(t)}*

*p^+^(t) = #{j = 0,1,…M; D_j_(t) ≥ D_0_(t)}/(M+1)*

*p^–^(t) = #{j = 0,1,…M; D_j_(t) ≤ D_0_(t)}/(M+1)*

where # signifies the number of elements in the set. The significance level for pointwise comparison, α_p_, was set 0.05. To compensate multiple comparison through all time points in the PETH, the global significant level α_g_ was also computed by adjusting the value to make error rate (number of false rejection of the null hypothesis) to be < 0.05, using the resampled data. The time points with *p*(t) < α_g_ (time point passing the global level) and *p*(t) < α_p_ (time point passing the pointwise level) were identified. A time segment consecutively passing the pointwise level and including at least one point passing the global level were determined as significant. For the theoretical justification and detailed explanation of the analysis, see Fujisawa et al., 2008 (see its section “Materials and Methods” and Supplementary Figure 4).

To analyze the individual and population dynamics during a locomotor task, continuous PETHs from a trial were made by stretching the time axis of instantaneous firing rate of neurons. The instantaneous firing rate was calculated by filtering the spike train with a Gaussian kernel (σ = 50 ms) and re-sampling the filtered signal at 100 Hz. To stretch the time axis, six alignment events *T*_i_ (*i* = 1…6) were defined as follows:

*T_1_ = T_2_ − 1.5 (s)*

*T_2_ = start timing (s)*

*T_3_ = T_2_ + median of preparatory duration (s)*

*T_4_ = T_3_ + median of response delay (s)*

*T_5_ = T_4_ + 1 s (s)*

*T_6_ = T_5_ + 1.5 s (s)*

And corresponding events t_i_ in each trial were defined as follows:

*t_1_ = t_2_ − 1.5 s (both rewarded and unrewarded trials)*

*t_2_ = start timing (both rewarded and unrewarded trials)*

*t_3_ = cue onset (both rewarded and unrewarded trials)*

*t_4_ = restart timing (rewarded trials), or t_4_ = t_3_ + median response delay (unrewarded trials)*

*t_5_ = t_4_ + 1 s (both rewarded and unrewarded trials)*

*t_6_ = t_5_ + 1.5 s (both rewarded and unrewarded trials)*

The instantaneous firing rate of the neurons was stretched along the time axis in a piecewise-linear manner to align each *t*_i_ with the corresponding *T*_i_, and averaged across the trials to construct the continuous PETH.

### Demixed Principal Component Analysis (dPCA)

For the population analysis of ACC neuronal activity, we used demixed principal component analysis (dPCA), recently developed by Kobak et al. (2016). The analysis allowed us to compress the dimensions of the standard PCA as well as extract the dependencies of the population activity on various aspects of the task. The details for this method are thoroughly explained by Kobak et al. (2016). Briefly, the trials were tagged based on two conditions: whether it was rewarded or not in the previous trial (condition *P*) or in the current trial (condition *C*). The population activity *X* was decomposed in a way analogous to the variance decomposition performed in ANOVA as follows:

$$\text{X}={\text{X}}_{\text{t}}+{\text{X}}_{\text{Pt}}+{\text{X}}_{C\,t}+{\text{X}}_{\text{PCt}}+{\text{X}}_{\text{noise}}=\sum_{\Phi }{\text{X}}_{\Phi }\,{\text{X}}_{n\,o\,i\,s\,e},$$

where *X*_t_, *X*_Pt_, *X*_Ct_, *X*_PCt_, and *X*_noise_ represent condition-independent, condition *P*-dependent, condition *C*-dependent, *P*-*C* interaction dependent, and noise terms. Then, dPCA finds separate decoder (*D*) and encoder (*F*) matrices for each *X*_Φ_, by minimizing the following loss function:

$$\text{L}=\sum_{\Phi }{∥{\text{X}}_{\Phi }-{\text{F}}_{\Phi }\,{\text{D}}_{\Phi }\,\text{X}∥}^{2}$$

Each component of the optimized *D*_Φ_ was designated as a demixed principal component (dPC). All of the resultant dPCs were ordered and numbered by the amount of explained variance. To avoid overfitting, dPCA was performed with regularization (Kobak et al., 2016). A cross-validation was performed to measure the accuracy of time-dependent condition-classification. Moreover, a shuffling procedure (100×) was used to assess whether the accuracy was significantly above chance. These processes were performed with the MATLAB scripts provided in Kobak et al. (2016)^2^.

## Results

### Behavioral Parameters During the Locomotor Task

**FIGURE 3:**
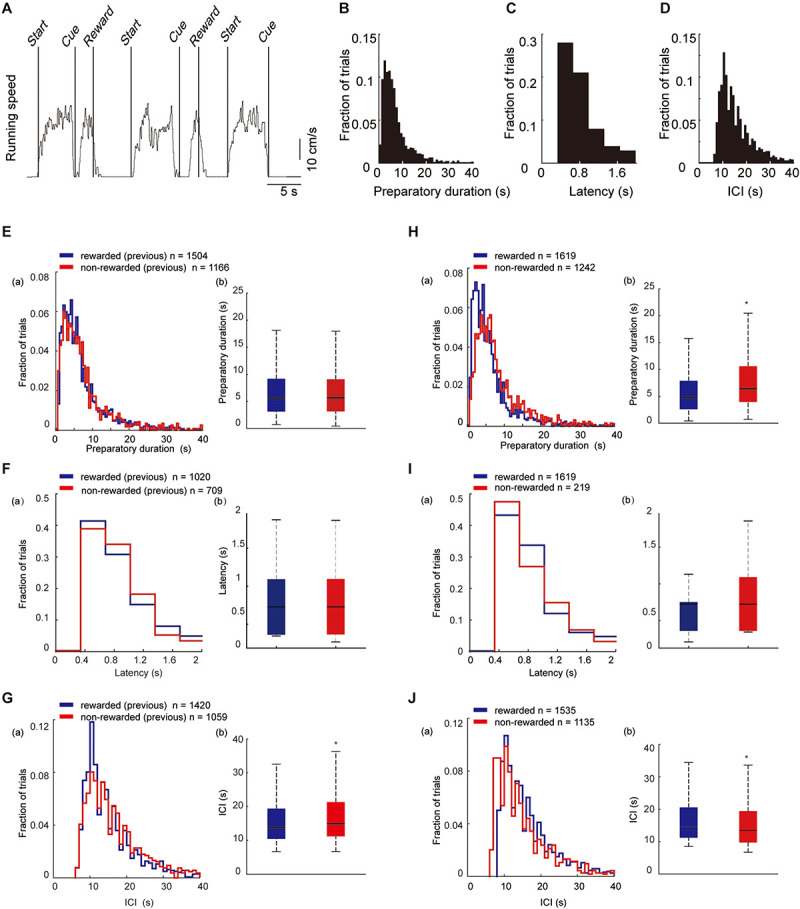
Behavioral parameters in trials with different behavioral outcomes. **(A)** A representative trace recording of locomotion velocity. *Start* indicates the beginning of the preparatory run; *Cue* indicates the time of visual-cue presentation; and *Reward* indicates the time of reward delivery. **(B–D)** Histogram of the duration of the preparatory run (*n* = 1,619). **(E)** Histogram of the latency to start the reward run after visual-cue presentation (*n* = 1,157). **(F)** Histogram of the duration of ICI (*n* = 1,535). **(E–G)** Histograms (a) and bar graphs (b) of the mean duration of the preparatory run, response latency, and ICI in trials that were preceded by a rewarded trial (blue) or by a non-rewarded trial (red). The duration of preparatory run **(E)** and latency **(F)** from first trials are not included in the analysis since they did not have a previous outcome. The ICI **(G)** between the first trial and the second trial were excluded as well. **(H–J)** Histograms (a) and bar graphs (b) of the mean duration of the preparatory run, latency, and ICI in rewarded (blue) and non-rewarded (red) trials. Statistical differences were calculated by two-sided Wilcoxon signed-rank test (*p < 0.05). (**E**b) *n* = 2,670 trials, *z* = −1.75, *P* = 0.08; (**F**b) *n* = 1826 trials, *z* = 0.57, *P* = 0.58; (**G**b) *n* = 2,479 trials, *z* = −4.47, *P* = 7.91E-6; (**H**b) *n* = 2,479 trials, *z* = −10.11, *P* = 4.95E-24; (**I**b) *n* = 1,834 trials, *z* = −0.20, *P* = 0.83; (**J**b) *n* = 2,670 trials, *z* = 7.65, *P* = 1.95E-14). In box plots, the central mark indicates the median and the bottom and top edges of the box indicate the 25th and 75th percentiles, respectively. Whiskers extend to the most extreme data points not considered outliers.

During the locomotor task, a trial was initiated when the mouse spontaneously started to run on the treadmill (preparatory run). Immediately after the mouse stopped running, a visual cue was presented for 2 s, a time window during which the animal had to restart running to obtain a reward (reward run). When the mice ran more than 1 s, sucrose solution was delivered from the spout (see section “Materials and Methods” for details). A representative trace of the ball velocity during the task is shown in Figure 3A. The mouse typically stopped running when it was rewarded with the sucrose water, and then continued to run for the next trial. The mean success rate (proportion of rewarded trials) was 52.7% ± 1.3% (mean ± SEM, 2,861 trials, 189 sessions, seven mice). The preparatory run was self-paced, and in the majority of the trials it lasted for several seconds (range 0.43–124.55 s, mean duration of all the trials: 8.72 ± 0.16 s, Figure 3B). For the reward run, mice typically began running again within 1 s after the visual cue was presented. The mean latency of the start of the reward runs after the cue presentation was 0.71 ± 0.01 s (Figure 3C). The mean duration of a single rewarded trial (from the start of the preparatory run to the timing of the reward delivery) was 8.67 ± 0.20 s. The inter-cue interval (ICI) duration (the duration of the cue presentation between two consecutive trials) was 19.19 ± 0.50 s (Figure 3D). The mean duration of a single session was 6.20 ± 0.04 min. There was no significant differences in the duration of one session or the proportion of rewarded trials between the sessions in a single day.

Next, we examined whether the outcome of the previous trial (previous outcome) had an effect on the behavioral parameters. There was no significant difference in the duration of the preparatory run between pre-rewarded and pre-non-rewarded trials (Figure 3E). Likewise, no significant differences were found in response latency (Figure 3F). However, ICI duration (Figure 3G) between the trials that were preceded by a rewarded or non-rewarded trial, was shorter in rewarded trials. We then examined whether there was a difference in behavioral parameters depending on the outcome of the current trial (current outcome). The duration of the preparatory run was significantly longer in non-rewarded trials (8.82 ± 0.25 s) compared with rewarded trials (6.93 ± 0.19 s, Figure 3H). There were no significant differences in response latency between the rewarded trials and non-reward trials in which the mice ran less than 1 s in the reward run (Figure 3I). However, the ICI duration after the non-rewarded trial was shorter than that after the rewarded trial (Figure 3J).

### Activity of Individual ACC Neurons During the Locomotor Task

**FIGURE 4:**
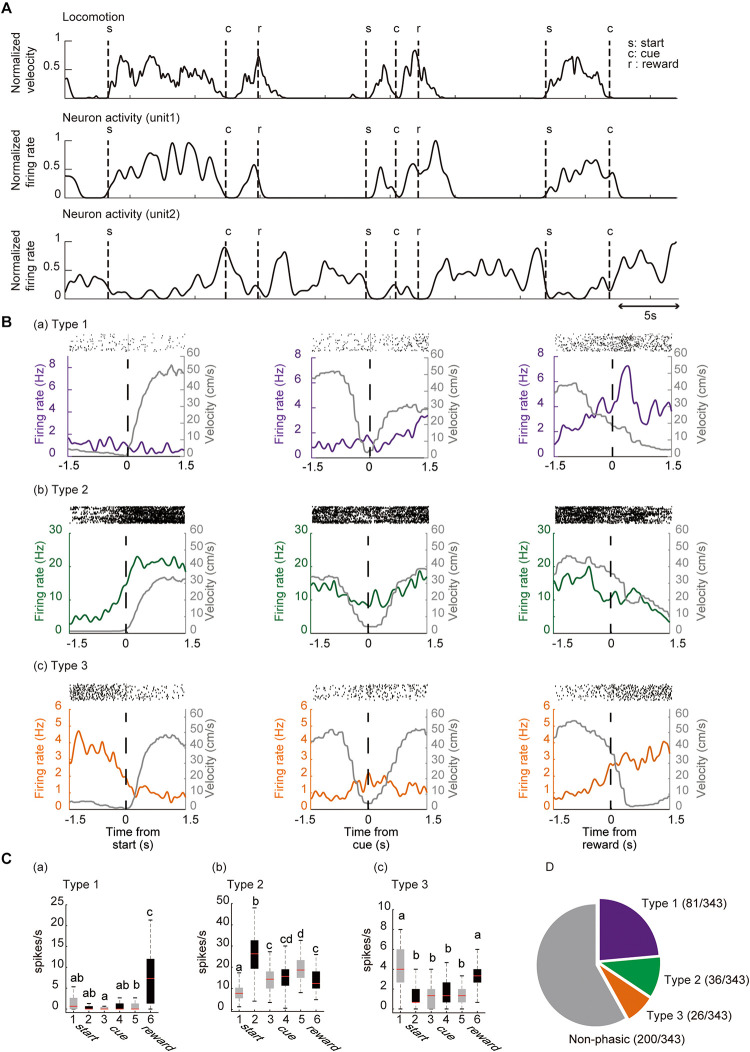
Categorization of locomotor task-related neurons. **(A)** Representative traces of locomotion velocity (upper panel) and the simultaneous recordings of two ACC units (middle and lower panel) during the locomotor task. **(B)** Spiking activity in representative units, categorized based on their phasic activity as Type 1 (a), Type 2 (b), and Type 3 (c) neurons. Spike raster plots for 45–60 consecutive trials are shown in the top panel of each peri-event histogram (PETH). The lower panels show the averaged firing rate and the running velocity (gray), 1.5 s before and after each key task event. Vertical dashed lines in each graph indicate the time of the key task event. **(C)** Box plot of the firing rate 1.5 s before start (1), 1.5 s after start (2), 1.5 s before cue (3), 1.5 s after cue (4), 1.5 before reward (5), 1.5 after reward (6) of each neuron. Gray and black boxes indicate the mean firing rate before and after the event, respectively. Examples of a Type 1 (a), Type 2 (b), and Type 3 (c) neuron. Different lower-case letters on each bar indicate a significant difference, *p* < 0.05 (Bonferroni tests after one-way ANOVA) between each other. **(D)** Summary of the number of ACC neurons in each category. In box plots, the central mark indicates the median and the bottom and top edges of the box indicate the 25th and 75th percentiles, respectively. Whiskers extend to the most extreme data points not considered outliers.

We isolated 343 units in the ACC from seven mice and analyzed their firing activity during the task performance. The activity of these neurons was analyzed around the key task events. The recorded neurons were classified by significant phasic activity around the key task events. Figure 4A shows a representative electrophysiological recording during the locomotor task from a pair of units. More than 40% of the recorded neurons (143 out of 343 neurons) were significantly modulated in at least one of the key task events. Since it has been shown in previous studies that ACC neurons are modulated by reward-related events (Cowen et al., 2012; Ma et al., 2014), we first termed 81 neurons (23.6%, 81/343) that were significantly modulated around the timing of the reward delivery as Type 1 neurons (Figures 4Ba,Ca). Thirty-six neurons (10.0%, 36/343) significantly increased their firing rate when the mouse started to run (Figures 4Bb, 5Cb); and they were termed Type 2 neurons. By contrast, the firing rate of 26 neurons (7.6%, 26/343) was significantly decreased when the mice started to run, and significantly increased when they stopped running; and they were termed Type 3 neurons (Figures 4Bc,Cc). These results indicate that the ACC contains neurons that encode the key elements (preparatory run, reward run, and reward) of the locomotor task. Significant changes in the firing rate after the cue presentation were observed in 24 recorded neurons (13 type-1 neurons, 7 type-2 neurons and 4 type-3 neurons). Approximately 60% of the recorded neurons (58.3%, 200/343) did not show significant phasic change in their firing rate around the key task events; thus they were classified as non-phasic neurons (Figure 4D).

**FIGURE 5:**
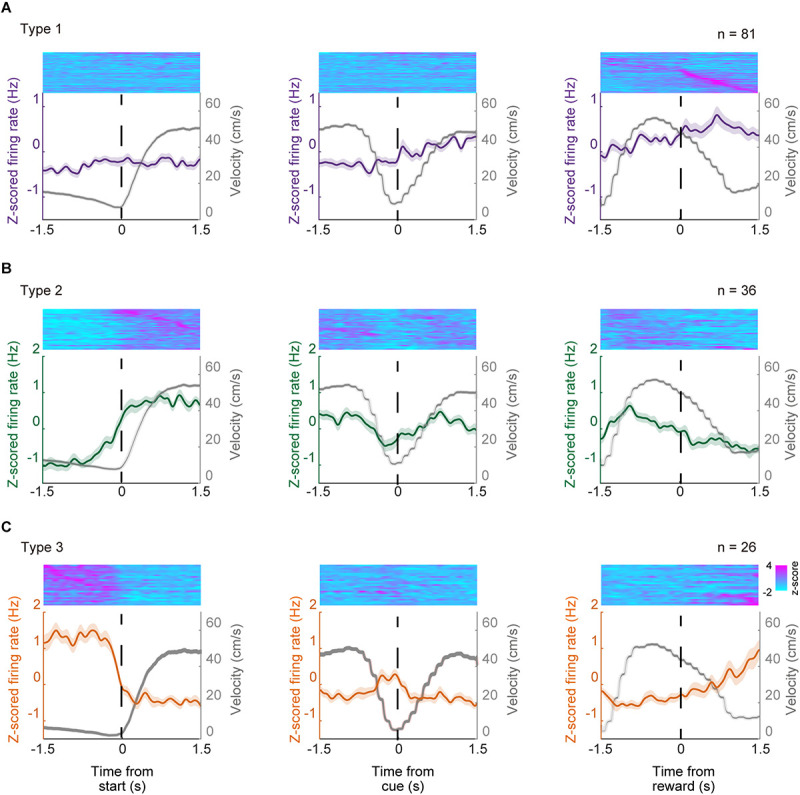
Population activity of type 1–3 neurons. Normalized *Z*-scored firing rates of phasic ACC neurons; type 1 neurons **(A)**, type 2 neurons **(B)**, and type 3 neurons **(C)**. Normalized firing rate of individual units are shown in the top panels whole PETH that of the mean *z*-scored firing rate of each neuron type and running velocity are shown in lower panels. Each unit were sorted according to the timing of the maximum value ( ±1.5 s) relative to the key task events. *Z*-scored activity profiles were obtained for each neuron by using the instantaneous firing rate for each unit, which was produced by convolving a Gaussian kernel with σ = 50 ms resampled at 100 Hz for each single trial.

Figure 5 shows the mean *z*-scored firing rate of each neuron type recorded for ± 1.5 s outside of the key task events, normalized relative to the peak firing rate during the task. These results indicate that these neuron types show phasic firing patterns at each related key event while individual neurons showed heterogeneous firing patterns even within the same neuron type while performing the task. We also examined the correlation between the firing rate of these phasic neurons and the running velocity by computing the Pearson’s correlation coefficient (r). The mean r values were 0.00 ± 0.02, 0.09 ± 0.03, −0.20 ± 0.03 for Type 1, Type 2, and Type 3 neurons, respectively (Supplementary Figure 1). There was a significant difference between mean r values of the three neuron types (Bonferroni tests after repeated measures one-way ANOVA, *p* < 0.01, Type 1 vs. Type 3, Type 2 vs. Type 3; *p* < 0.05, Type 1 vs. Type 2). There were 7 neurons (4.9% of phasic neurons, 4 Type-1 neurons, 3 Type-2 neurons, 0 Type-3 neurons) that showed significant positive correlation (*r* > 0.30, *p* < 0.05) with running speed while 11 neurons (7.7% of all phasic neurons, 1 Type-1 neuron; 2 Type-2 neurons; 8 Type-3 neurons) showed significant negative correlation *(r* < −0.30, *p* < 0.05).

**FIGURE 6:**
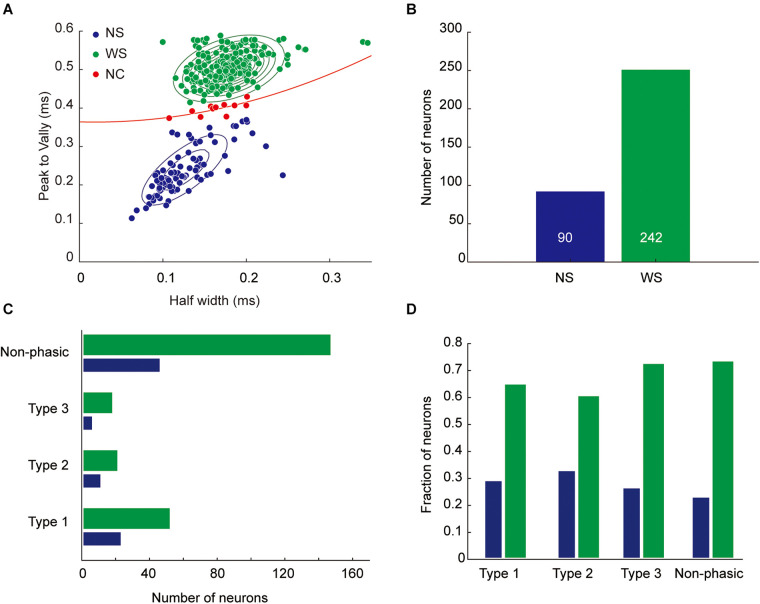
Classification of recorded neurons based on spike waveforms. **(A)** Units were classified as wide-spike (WS) neurons (green circles, putative pyramidal cells) or narrow-spike (NS) neurons (blue circles, putative interneurons) based on the shape of the spike waveform. For the classification, a Gaussian mixture model was fitted to the units. Units close to the separatrix (dashed red line) with low classification confidence (*p* > 0.05, red circles, *n* = 10) were not classified (non-classified, NC). **(B)** Number of neurons in each class. **(C)** Number of classified neurons in each task-related category. **(D)** Proportion of classified neurons in each task-related category.

Based on their spike waveform features, we were able to classify most of these neurons into WS neurons (*n* = 242; half-valley width, 179.0 ± 2.0 μs) and NS neurons (*n* = 90; half-valley width, 124.7 ± 3.7 μs; Figures 6A,B). The former were considered as putative pyramidal cells, and the latter as putative interneurons. These results are in agreement with previous unit recording studies in the rodent medial PFC (Fujisawa et al., 2008; Kim et al., 2016). There were no significant differences in the proportion of WS and NS neurons between each phasic category (Chi-square test, *p* = 0.096, Figures 6C,D).

**FIGURE 7:**
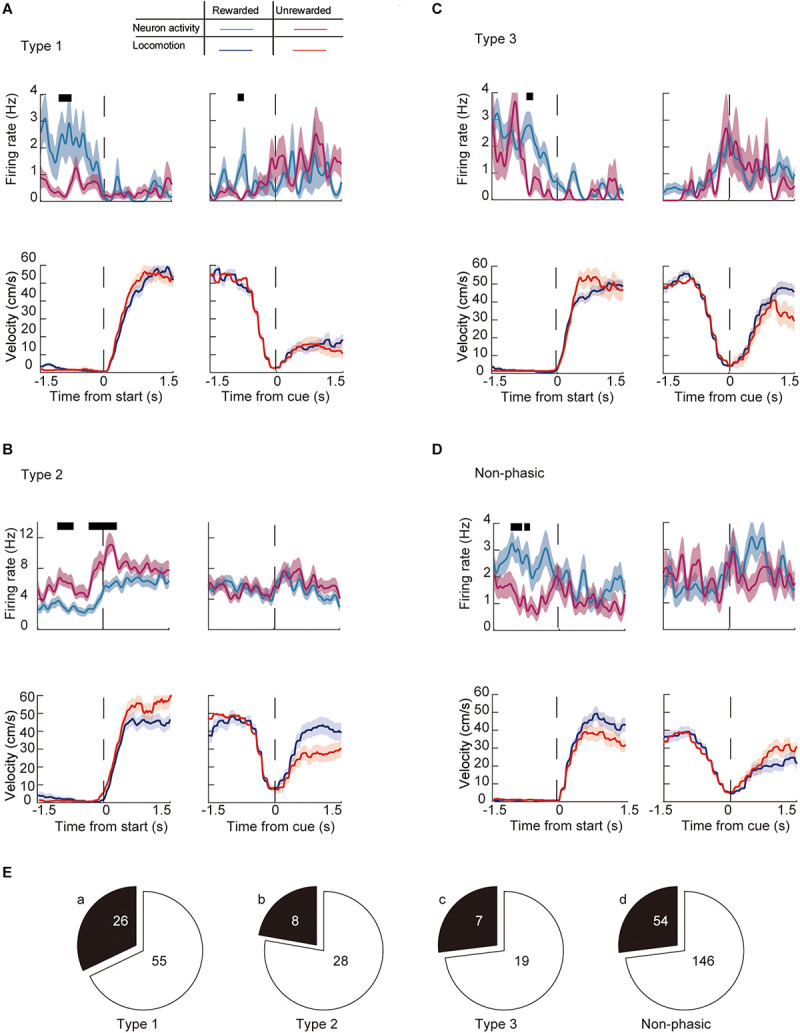
Modulation of the firing activity of ACC neurons around the key task by behavioral outcomes in the previous trial. Examples of the running velocity (upper panels) and neuronal activity (lower panels) of **(A)** Type 1, **(B)** Type 2, **(C)** Type 3, and **(D)** non-phasic neurons ± 1.5 s from the start of the preparatory run, and cue presentation during previously rewarded (blue lines for the running velocity, cyan for the firing rate) and previously non-rewarded trials (red lines for the running velocity, magenta for the firing rate). Thick black lines in each panel represent significantly different time segments (*P* < 0.05; see section “Materials and Methods”). **(E)** Number of significantly modulated (a) Type 1, (b) Type 2, (c) Type 3, and (d) non-phasic neurons. Shaded areas in each trace indicate SEM.

We then examined whether the activity of individual neurons during a trial changes depending on the outcome (i.e., if the animal received a reward or not) of a previous trial (previous outcome). Figure 7 shows a representative recording of phasic and non-phasic neurons that fired differently around the start and end of the preparatory run based on different previous outcomes. Almost 30% of phasic neurons (28.6%, *n* = 41/117) showed a significantly different firing rate between trials that were preceded by a rewarded trial compared with those that were preceded by a non-rewarded trial. Specifically, 32.1% of Type 1 (*n* = 26, Figures 7A,E), 22.2% of Type 2 (*n* = 8, Figures 7B,E), and 26.9% of Type 3 (*n* = 7, Figures 7C,E) neurons were differentially modulated by the previous outcome. Furthermore, 27.0% of the non-phasic neurons (*n* = 54, Figures 7D,E) were differentially modulated by the previous outcome. The number of differentially modulated phasic and non-phasic neurons was higher around the start of the preparatory run (*n* = 84, 24.5%) than the cue presentation (*n* = 12, 3.5%, Table 1). These results indicate that previous outcomes have modulated the activity of a fraction of the ACC neuronal population, particularly during the early part of the trial.

**TABLE 1:** Number of neurons modulated by behavioral outcome.

	Previous outcome		Current outcome	
Events	Start	Cue	Start	Cue
Type 1 (*n* = 81)	24	2	10	21
Type 2 (*n* = 36)	6	2	7	19
Type 3 (*n* = 26)	7	0	1	20
Phasic (*n* = 143)	37	4	18	60
Non-phasic (*n* = 200)	47	8	31	58
Total (*n* = 343)	84	12	49	118

**FIGURE 8:**
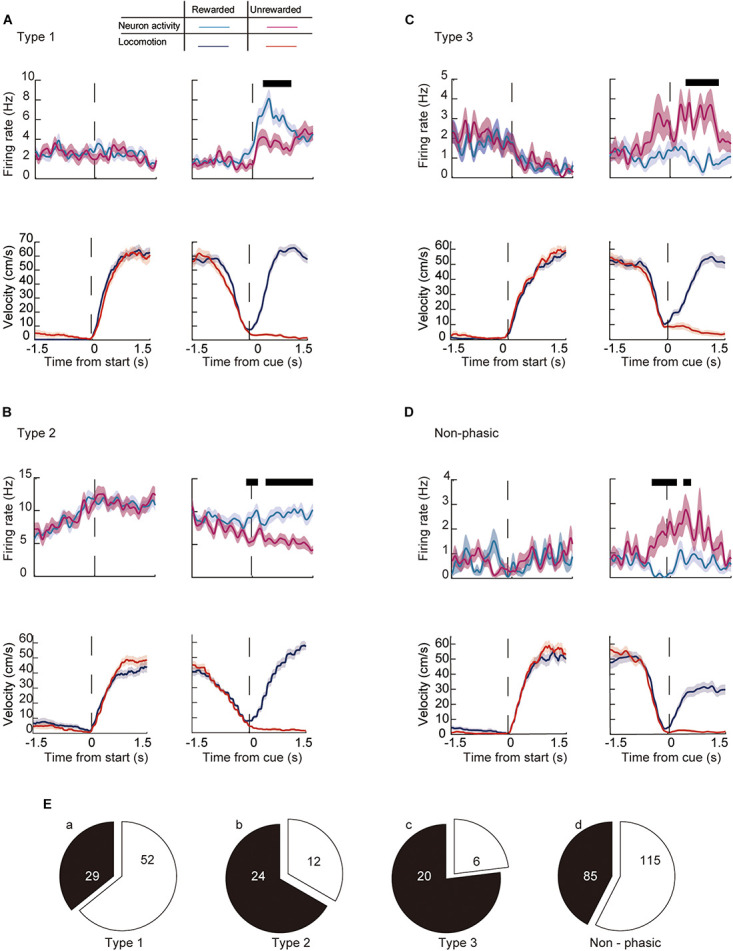
Modulation of the firing activity of ACC neurons around the key task by behavioral outcome in the current trial. Examples of the running velocity (upper panels) and neuronal activity (lower panels) of **(A)** a Type 1, **(B)** a Type 2, **(C)** a Type 3, and **(D)** a non-phasic neuron around the start and cue presentation, during rewarded (blue lines for the running velocity, cyan for the firing rate) and non-rewarded trials (red lines for the running velocity, magenta for the firing rate). Thick black lines in each panel represent significantly different segments (*P* < 0.05; see section “Materials and Methods”). **(E)** Number of significantly modulated (a) Type 1, (b) Type 2, (c) Type 3, and (d) non-phasic neurons. Shaded areas in each trace indicate the SEM.

We then analyzed whether the activity of individual ACC neurons differ between rewarded trials and non-rewarded trials while the animal performed the task (current outcome). Figure 8 shows representative recordings of individual neurons in each category around the start and end of the preparatory run. The majority of phasic neurons (57.4%, *n* = 73/143) showed a significantly different firing rate in rewarded compared with unrewarded trials. Among them, 35.8% of Type 1 (*n* = 29, Figure 8A), 66.7% of Type 2 (*n* = 24, Figure 8B) and 76.9% of Type 3 (*n* = 20, Figure 8C) neurons were differentially modulated between the two conditions (Figure 8E). In addition, 42.5% of non-phasic neurons fired significantly differently in rewarded and non-rewarded trials (*n* = 85, Figures 8D,E). Compared with previous outcomes, a smaller number of neurons were modulated by the current outcome at the start of the preparatory run (Table 1). However, the number of modulated phasic and non-phasic neurons by current outcomes were higher around the cue presentation (*n* = 118, 34.4%) compared with the start of the preparatory run (*n* = 49, 14.3%, Table 1). These results indicate that a fraction of the ACC neuronal population is active in rewarded or non-rewarded trials around the key task events.

### Population Activity of ACC Neurons During the Locomotor Task

**FIGURE 9:**
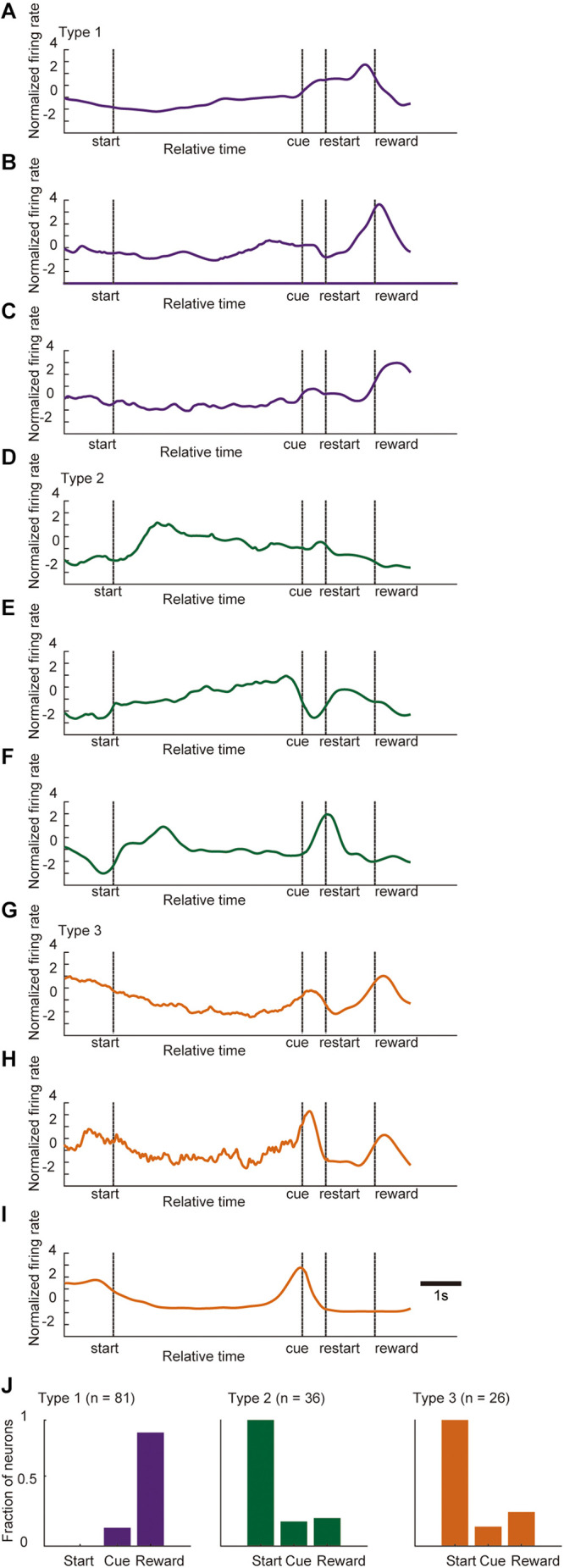
Normalized activity of phasic neurons during a trial. Examples of the normalized and time-stretched firing rate change (see section “Materials and Methods” for details) in Type 1 neurons **(A–C)**, Type 2 neurons **(D–F)**, and Type 3 neurons **(G–I)** throughout the task. **(J)** Bar graphs of the fraction of phasic neurons in each category that showed significant change in firing rate in each key task event (start, cue, and reward). Statistical differences, *p* < 0.05 (Bonferroni tests after one-way ANOVA).

**FIGURE 10:**
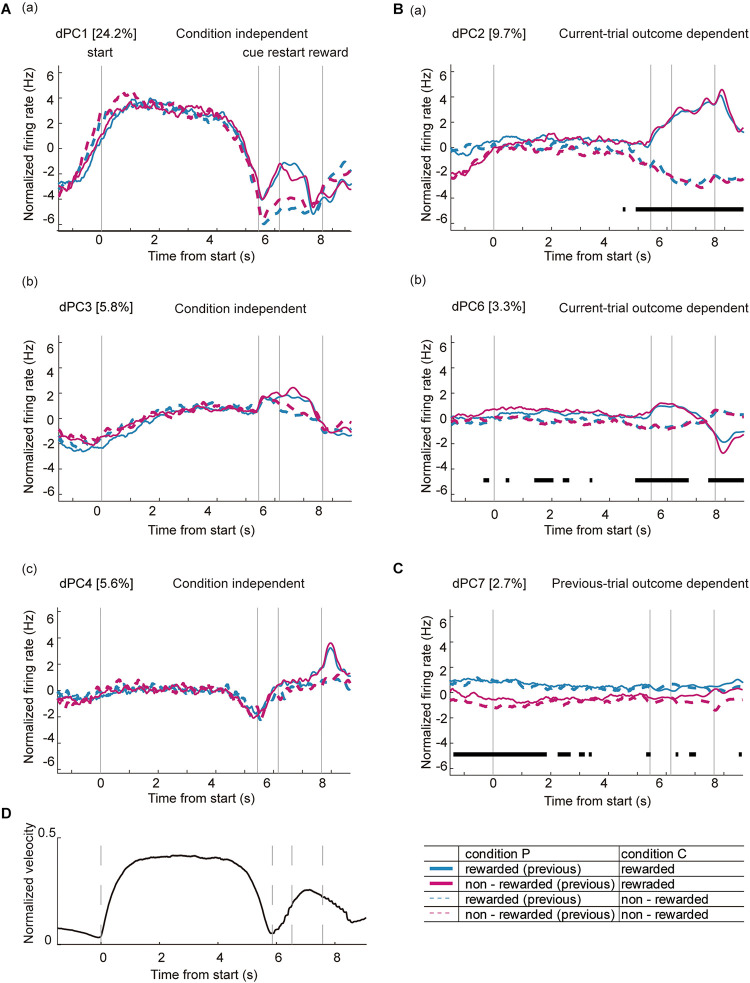
Demixed principal component analysis of ACC neurons during the locomotor task. Time course of dPCs during the locomotor task: **(A)** condition-independent components, **(B)** components dependent on the behavioral outcome of the current trial (current-trial-outcome-dependent), and **(C)** components dependent on the behavioral outcome of the previous trial (previous-trial-outcome-dependent). Dashed lines indicate the timing of each key task event (start, cue, and reward). Variance of each dPCs are shown as percentage. Thick black lines in panels **(B,C)** indicate that the timing of the respective condition (rewarded or non-rewarded) can be reliably extracted from the single-trial activity. **(D)** Averaged velocity of locomotion during a single trial in all of the animals. The locomotion velocity in all trials were averaged, normalized, and time-stretched in a similar way to the dPCA analysis of neurons.

As indicated in Figure 5, there was a complex dynamic of ACC activity around the key task events. Moreover, some of the phasic neurons showed heterogeneous and complex patterns during the task. Figure 9 shows several examples of the normalized firing rate change in Type 1 (Figures 9A–C), Type 2 (Figures 9D–F), and Type 3 (Figures 9G–I) neurons throughout the task. A fraction of neurons in all of the categories of phasic neurons showed significant phasic change in firing rate at multiple key task events (Figure 9J). Furthermore, a significant fraction of phasic and non-phasic neurons showed differential firing depending on the behavioral outcome in previous and current trials. Therefore, to determine the representative activity of the ACC at the population level during the locomotor task, we applied demixed principal component analysis (dPCA), a recently developed dimensional reduction technique (Kobak et al., 2016). Principal component analysis (PCA) has been widely used to extract a set of principal components (PCs) that are computed to maximize the amount of explained variance. However, conventional PCA does not take information about task parameters into account and therefore the mixed selectivity of the neuronal population remains unseparated. The dPCA is effective for identifying the main components and factors underlying the variance of neuronal signaling, and relate the activity to various task parameters (Kobak et al., 2016; Rossi-Pool et al., 2017). For an unbiased analysis of normalized activity, all of the recorded neurons, including those of non-phasic neurons in all of the recorded trials, were included. The trials were tagged based on four conditions, and whether the mice were rewarded or not in the previous trial (condition P) and current trial (condition C). The first demixed principal component C (dPC1) accounted for 24.2% of the total explained variance and was extracted as a condition-independent component. dPC1 exhibited an increase at the start of the preparatory run, a gradual decrease before the cue presentation, and a small increase before the reward presentation (Figure 10Aa). The time course of the eigenvector resembled that of the locomotion velocity (Figure 10D). Other condition-independent dPCs included the third (dPC3, 5.8%) and fourth (dPC4, 5.6%) components (Figures 10Ab,c). Among these dPCs, dPC3 gradually increased during the preparatory run and showed further increase after the cue presentation in both rewarded and non-rewarded trials while dPC4 decreased toward the end of the preparatory run and showed an increase after the cue presentation. The second dPC (dPC2), which accounted for 9.7% of the total explained variance, was extracted as a condition-C-dependent component (Figure 10Ba). In rewarded trials, it gradually increased after the cue presentation and peaked after the reward delivery. In this component, significant tuning of the neurons could be observed just before the end of the preparatory run/cue presentation until the reward delivery. The other major condition-C-dependent component was dPCA6, which accounted for 3.3% of the total explained variance (Figure 10Bb). In this component, significant tuning of the neurons in rewarded trials could also be observed just before the end of the preparatory run/cue presentation and after the restart (onset of reward run). Additionally, the significant tuning reappeared after the reward delivery. Finally, among the condition-P-dependent components, the largest was the seventh component (dPC7, 2.7%), which decreased at the start of the preparatory run but only in trials following non-rewarded trials (Figure 10C). Such significant tuning of neurons was observed before the onset of the preparatory run and lasted throughout the initial part of the preparatory run. These results indicate that the ACC neurons encode the temporal information of locomotion, including initiation and termination, as well as the retrospective and prospective behavioral outcome while performing the task. Furthermore, it appears that the previous outcome continues to be encoded at the start of the preparatory run, while the representation of the behavioral outcome of the current trial emerges just before the animal stops the preparatory run, and the cue for the reward is presented. These findings suggest that a population of ACC neurons encode the motor aspect, including the timing of the execution and termination of the movement, as well as past and present behavioral outcomes while performing the task.

## Discussion

The present study showed that a significant fraction of the ACC neurons were involved in the initiation and termination of the locomotion, as well as in the reward response behavior. Although the individual neurons exhibited heterogenous and mixed firing patterns, the population activity of the ACC neurons primarily represented the time course of the locomotor task. Other major components extracted from the population activity were modulated during the visual-cue presentation and reward period, depending on the behavioral outcome.

### ACC Activity Encodes Locomotion

The main finding of this study is that the ACC activity mainly represents locomotion during motivated reward-based behavior which requires repetitive running. This is in agreement with previous studies indicating that the ACC is involved with motor control and sensory monitoring. Namely, it has been shown that the dorsal part of the medial PFC has a strong connection with the motor and pre-motor cortical areas, which project their axons directly to the spinal cord where the basic locomotor centers are located (Grillner and El Manira, 2020). In the present study, the activities of more than 20% of the recorded neurons were significantly modulated at the onset and/or end of locomotion. Fifty percent of these neurons (Type 2 neurons) increased their firing rate at the onset of the preparatory run, and then decreased at the end of the preparatory run or after the reward delivery, suggesting that they are encoding the proactive locomotor element of the task. By contrast, Type 3 neurons were active during the ICIs and were mostly inhibited while the animal was running suggesting that they encode the inactive element. 12.6% of phasic neurons recorded in this study showed firing activity either positive or negative correlated with the running speed. In previous studies, a similar phasic pattern was observed in other motor tasks such as pressing and releasing a lever (Narayanan and Laubach, 2006), and poking their nose into a port (Murakami et al., 2017). In addition, the inactivation of dorsomedial PFC reduced the firing activity of the motor cortex neurons while holding, but not releasing, the bar at the end of the task (Narayanan and Laubach, 2006). Thus, the ACC may also be involved in top-down control of locomotion while integrating inputs from the limbic system. Murakami et al. (2017) showed that individual dorsomedial PFC neurons encoded the start of the movement by increasing or decreasing their firing rate. Although there was no delay/waiting period imposed in the locomotor task in this study, the Type 3 neurons, which were active during the ICIs, may constitute a part of the circuit that controls the timing of the motor behavior.

At the populational level, the first component of the dPCA represented the time course of locomotion that was independent of behavioral outcomes. This indicates that the ACC mainly encodes the timing of locomotion, specifically the initiation, maintenance and termination of the movement. Previous studies examining the activity of medial PFC neurons while rats performed a spatial navigation task showed that a significant proportion of the neurons responded to the direction or velocity of running (Jung et al., 1998; Cowen et al., 2012). In these studies, the neuronal activity was correlated with multiple behavioral task parameters, such as the position of the animal in the maze or its direction. In the current study, ACC activity was recorded in a head-immobilized mouse without spatial information; thus the neurons may have shown a less complex firing pattern.

During the locomotor task, the mouse spontaneously initiated and stopped running before the reward run. In most of the rewarded trials, the latency to restart was shorter than 1 s, suggesting that the animals were prepared for the next action before they ended the preparatory run. The second and sixth dPC component (dPC2 and dPC6, respectively) were dependent on the resulting behavioral outcome. These components showed significant tuning of the neurons just before visual-cue presentation, indicating that ACC activity could be differentially modulated by the behavior to commence the reward run. This is consistent with the pattern of individual ACC neuron activity because more than one-third of the recorded neurons showed differential firing depending on the prospective behavioral outcome. Interestingly, mice ran significantly longer in non-rewarded trials than in rewarded trials, indicating that the timing of the termination of the preparatory run could be associated with the next motor decision during the task. It has been indicated by previous results that the medial PFC is involved in maintaining a set of motor decisions while the animal is engaged in goal-directed behavior (Narayanan and Laubach, 2006; Murakami et al., 2017). Taken together, these results indicate that the ACC neuronal population strongly represents the motor aspect of the reward-based task.

### ACC Activity May Represent the Context of Locomotion

The ACC has projections to the amygdala and lateral hypothalamus, both of which are important for reward-seeking and predator-evasion behaviors (Reppucci and Petrovich, 2016; Stuber and Wise, 2016). In a recent study, it was shown that activation of the ACC suppresses the fear response/freezing by modulating the activity of neurons in the basolateral nucleus of amygdala (Jhang et al., 2018). These structures are thought to be playing an important role in controlling locomotion based on context or executing innate behaviors (Ferreira-Pinto et al., 2018). The ACC may process temporal information related to locomotion in a top-down manner while executing motivated behaviors. Based on the dPC2 and dPC6 being the main components in the dPCA, the ACC neurons may modulate the reward-based behavior while encoding the movement. These results may also reflect the level of attention of the animal during the motor task. The ACC has strong connections with the cortical and subcortical areas involved in visual processing, such as the visual cortex and the superior colliculus (Zhang et al., 2014). Recent studies have suggested top-down modulation of visually guided behaviors (Leinweber et al., 2017; Hu et al., 2019). Leinweber et al. (2017) showed that in a head-fixed mouse navigating through a virtual reality arena, 30% of the axons projecting from ACC and M2 to the primary visual cortex (V1) are activated before the mouse starts running. Interestingly, both the ACC axons and visual cortex neurons were activated when the animal was running in the dark (Niell and Stryker, 2010; Leinweber et al., 2017). Moreover, it has been shown that locomotion alone or activating the ACC enhances the visual response in V1 and the performance of the visual behavior (Niell and Stryker, 2010; Hu et al., 2019). This suggests that the information of movement in the ACC is involved in elevating the activity levels in V1 and SC and contributing to the modulating the level of attention during goal directed behaviors. In the present study, we did not examine simple visual responses in this task, therefore we cannot dissociate the visual-cue related activity in the ACC. However, there were fractions of ACC neurons that showed phasic activity after the cue presentation and an increase was observed in condition independent components (dPC3 and dPC4) that indicate the visual information while performing this task may also be represented in this neuronal population. It would be of great interest to examine how this information processed in the ACC will influence the decision making with locomotor behavior by using a more complex behavioral task that requires discrimination of visual cues and active choice of actions.

### Encoding the Reward Signals During the Locomotor Task

The ACC Type 1 neurons increased their firing rate around the time of the reward delivery. This is in agreement with previous rodent studies (Cowen et al., 2012; Ma et al., 2014). Together with reports studying free-moving rats, the dPC2 and dPC6 results from this study support the theory that ACC neurons encode reward- and reward-expectation-related signals (Bryden et al., 2011; Cowen et al., 2012; Ma et al., 2014). Further, the ACC of mice has been shown to modulate the VTA, which is involved in reward and reward prediction (Schultz et al., 1997), thus influencing the motivation to pursue a reward (Elston and Bilkey, 2017). Recently, it was found that the dopaminergic neurons of head-fixed mice navigating through a virtual maze were modulated not only by reward and reward prediction, but also by the reward history (whether the previous trial was rewarded) and motor kinematics of the animal (Engelhard et al., 2019). Therefore, it is plausible that ACC representations of the motor- and reward-related aspects of the locomotor task are transmitted to the VTA to modulate the dopaminergic neurons during the motivational behaviors.

### Monitoring Behavioral Outcome Signal During Locomotion

Interestingly, the dPCA identified a component dependent on whether the mouse was rewarded or not in the previous trial (dPC7). These two different conditions could be reliably extracted in the beginning (around 2 s) of the preparatory run, indicating that ACC activity may be affected by experience even if locomotion was initiated for the next trial. Differential activity depending on the previous outcome was observed in one-fifth of the ACC neurons recorded, particularly at the beginning of the trial. It has been shown that in rats performing a nose-poke task, error-related signals were represented by ACC neurons (Totah et al., 2009). Previous studies in monkeys examining ACC activity during motivation-driven tasks showed that these neurons represented the behavioral outcome from past trials (Kawai et al., 2015), and were activated by negative outcomes such as reward omission (Ito et al., 2003; Matsumoto et al., 2003; Quilodran et al., 2008). Further, a recent study showed that perturbation of the ACC alters the timing of the action based on the history of the reward rate (Khalighinejad et al., 2020), suggesting that it is involved in determining future motor behavior using information from past outcomes. Our present results indicate that ACC neurons carry-over the information of past behavioral outcomes to the next trial, especially at the start of the preparatory run.

## Data Availability Statement

The raw data supporting the conclusions of this article will be made available by the authors, without undue reservation.

## Ethics Statement

The animal study was reviewed and approved by the Ethical Committee of Animal Experiments at the University of Toyama.

## Author Contributions

HNm designed the research. SR and HNm carried out the experiments. YT, MF, and HNm constructed the behavioral apparatus and controlling software. SR, JM, and HNm analyzed the data. HNm, SR, JM, YT, HNj, and TO wrote the manuscript. All authors contributed to the article and approved the submitted version.

## Conflict of Interest

The authors declare that the research was conducted in the absence of any commercial or financial relationships that could be construed as a potential conflict of interest.

## Footnotes

1 http://fmatoolbox.sourceforge.net/

2 http://github.com/machenslab/dPCA
