# Supplementary material for: Neuronal Representation of Locomotion During Motivated Behavior in the Mouse Anterior Cingulate Cortex
Source: Front Syst Neurosci. 2021 Apr 29;15:655110. doi: 10.3389/fnsys.2021.655110 (PMC8116624; doi:10.3389/fnsys.2021.655110)
Supplement: Supplementary file 1 [file Data_Sheet_1.PDF]

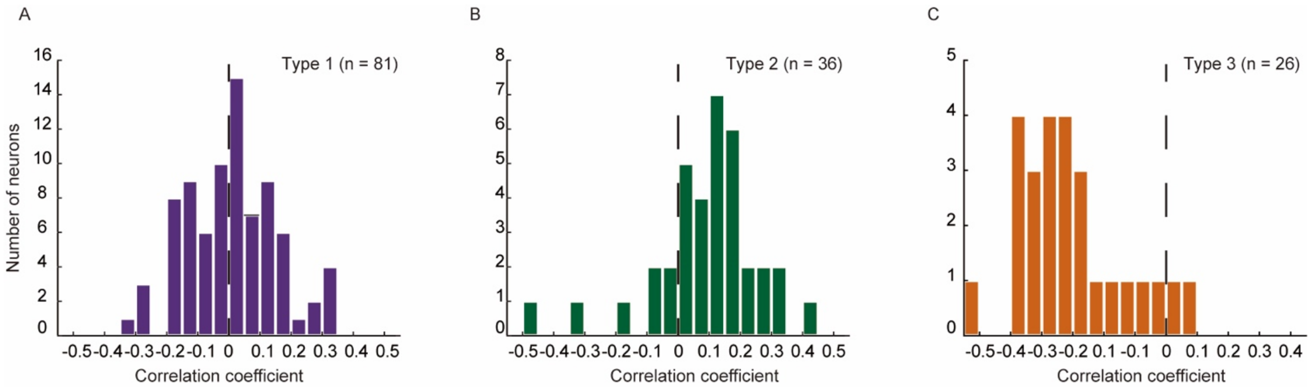


**Supplementary Figure 1.** Population histogram of the correlation coefficient (r) between firing rate and running velocity for Type 1 (A), Type 2 (B) and Type (C).
